# Supplementary material for: Thrombocytosis and abnormal liver enzymes: A trigger for investigation of underlying malignancy
Source: PLoS One. 2022 Apr 28;17(4):e0267124. doi: 10.1371/journal.pone.0267124 (PMC9049564; doi:10.1371/journal.pone.0267124)
Supplement: Attachment — Submitted filename: Response to Reviewers.docx [file pone.0267124.s002.docx]

.Journal Requirements

Question 1:

The manuscript has been edited to meet PLOS ONE’s style requirements. In addition, all figures were checked through PACE and improved files were submitted.

Question 2 and 3:

This was a retrospective study of routine patient data held within the NHS databases. Such studies do not require Medical Ethics approval in the UK as advised by Integrated Research Application System (<https://www.myresearchproject.org.uk/>).

The use of NHS data requires approval under Information Governance legislation in the UK, the local Caldicott Guardian approval was applied for and granted (letter attached in supporting information).

Question 4:

a) First author, LCG, received financial support from University of Dundee while undertaking the study as part of a Student Vacation Scholarship.
b) The funders had no role in study design, data collection and analysis, decision to publish, or preparation of the manuscript.
c) No authors received a salary from any funders
d) Other than author LCG’s Student Scholarship from University of Dundee, the remaining authors received no specific funding for this work.

Question 5:

a) None of the authors are employed by a commercial entity. Author ED has received unrestricted education grants from Siemens and Abbot unrelated to this paper.

b) Revised Competing Interests statement included in Cover Letter

Question 6:

The minimal underlying data set has been supplied in Supporting Information

Question 7:

All references were reviewed. An error in reference 4 was corrected.

Comments from Reviewers

*Reviewer #1: 
“Analysis of the validation cohort accepted cancer diagnoses if already present at time of request”. It may me think if this would be a bias and I would like a comment about it.*

Text has been adjusted to explain this – the validation cohort was taken from a random 6-month period of all blood requests across the health board with no information regarding reason for request. Therefore, it was not known how many previously abnormal results these patients had had and over what time frame and so all diagnoses were counted.

*Was there a trend in the number of platelets in patients with cancer? Do patients with advanced cancer had more platelets? Maybe, due to the retrospective analysis there is data about it.*

Text has been adjusted to reflect this – no trend was found.

*Reviewer #2: 
It would be worth clarifying if the control group had the same liver enzyme abnormality as the thrombocytosis group or whether this was a mixed bag of enzyme abnormalities.*

Text has been adjusted to reflect this – the control group did select a mixed group of enzyme abnormalities as they were randomly selected using only the parameters of age and sex.

*The cut off of platelet count of 400 is an arbitrary reference range level for the laboratory. With so many values being in the 400 to 450 range, were other values explored such as 350 or 375?*

I thank the reviewer for the comment as this would be beneficial to explore in the future. No other reference range was investigated in this study.

*On line 198 this refers to figure 3 not figure 2
On line 208 this refers to figure 4 not figure 3*

These errors have been corrected in the text.

*Table 2 could be rearranged in order site of metastases is below rather than alongside the site of the advanced cancers.*

The format of Table 2 has been rearranged.
