# Supplementary material for: Thrombocytosis and abnormal liver enzymes: A trigger for investigation of underlying malignancy
Source: PLoS One. 2022 Apr 28;17(4):e0267124. doi: 10.1371/journal.pone.0267124 (PMC9049564; doi:10.1371/journal.pone.0267124)
Supplement: Attachment — Submitted filename: Response to Reviewers 03_22.docx [file pone.0267124.s003.docx]

Response to Reviewers

**Journal Requirements**

*Please review your reference list to ensure that it is complete and correct. If you have cited papers that have been retracted, please include the rationale for doing so in the manuscript text, or remove these references and replace them with relevant current references. Any changes to the reference list should be mentioned in the rebuttal letter that accompanies your revised manuscript. If you need to cite a retracted article, indicate the article’s retracted status in the References list and also include a citation and full reference for the retraction notice.*

This requirement at the last review and I believe I have responded to this appropriately. If you believe that the references still require attention I would be very grateful for any further direction you can give so this can be resolved.

**Comments from Reviewers**

**Introduction: line ≠69: “Thrombocytosis is the term given to an overproduction of platelets…” Megakaryocyte proliferation is not the only cause of thrombocytosis as it can also result from decreased platelet sequestration (as in the case of asplenia, for example). Therefore, the sentence should be rewritten. One option of modification is: thrombocytosis is the presence of elevated platelet count… or the word ‘usually’ should be added to the existing sentence.*

The paper has been amended with this suggested change.

**Introduction: line ≠77 & 78: tuberculosis is written separately from bacterial causes as if it were not a bacterial infection. It should be modified like: The most common clinical scenarios in which reactive thrombocytosis occurs include bacterial infections including tuberculosis…*

The paper has been amended with this suggested change.

**Regarding exclusion criteria, was there any consideration of conditions that could potentially cause raised alkaline phosphatase such as growth healing fractures, osteomalacia, hyperparathyroidism, and hyperthyroidism? I would like to hear about this.*

In response to this query, exclusion criteria were not applied for patients with other causes of raised alkaline phosphatase. This was not considered as patients were being investigated for liver disease through intelligent liver function testing (iLFT). ALP is incorporated within the initial liver panel of iLFT and so excluding patients with raised alkaline phosphatase would limit the number of patients that had the remaining cascade of tests performed, including platelet count. However, I would like to thank the reviewer for the comment as this may be of use in future if the diagnostic tool is used out with iLFT.
